# Multimorbidity and associated factors in the adult Indigenous population living in villages in the municipality of Aracruz, Espírito Santo, State, Brazil

**DOI:** 10.1590/0102-311XEN135323

**Published:** 2025-02-07

**Authors:** Hully Cantão dos Santos, José Geraldo Mill

**Affiliations:** 1 Universidade Federal do Espírito Santo, Vitória, Brasil.

**Keywords:** Health of Indigenous Peoples, Multimorbidity, Chronic Disease, Prevalence, Salud de Poblaciones Indígenas, Multimorbilidad, Enfermedad Crónica, Prevalencia

## Abstract

Multimorbidity is associated with negative effects on the health of individuals, increasing the complexity of health care. This study aimed to determine the prevalence of multimorbidity and associated factors in the adult Indigenous population living in villages in Aracruz, Espírito Santo State, Brazil. This is a cross-sectional study using data from the project called *Assessment of the Prevalence and Severity of Chronic Diseases in the Indigenous Population of Espírito Santo State*. Data were collected from 2020 to 2022. Multimorbidity was defined as the presence of two or more chronic morbidities in a group of eight morbidities. As a measure of association, the prevalence ratio (PR) and its 95% confidence interval (95%CI), calculated by Poisson regression with robust variance, in crude models and models adjusted for covariates were used. The prevalence of multimorbidity was 52.1% (95%CI: 49.1-55.2), being significantly higher among women (PR = 1.47; 95%CI: 1.29-1.67), those aged ≥ 40 years (40-59 years: PR = 1.49; 95%CI: 1.28-1.73; ≥ 60 years: PR = 1.85; 95%CI: 1.55-2.20) and lower for individuals with higher education (PR = 0.65; 95%CI: 0.47-0.89). The prevalence of multimorbidity in the Indigenous population living in villages in Espírito Santo State was higher than that found in other studies in the general Brazilian population. There was association between the presence of multimorbidity and sex, age and education level.

## Abstract (es)

La multimorbilidad se asocia con efectos negativos sobre la salud de los individuos, lo que aumenta la complejidad de la asistencia sanitaria. Nuestro objetivo fue determinar la prevalencia de multimorbilidad y factores asociados en la población indígena adulta residente en Aracruz, Espírito Santo, Brasil. Se trata de un estudio transversal realizado con los datos del proyecto *Evaluación de la Prevalencia y Gravedad de Enfermedades Crónicas en la Población Indígena de Espírito Santo*. La recopilación de datos se realizó entre el 2020 y el 2022. La multimorbilidad se definió por la presencia de dos o más morbilidades crónicas en un grupo de 8 morbilidades. Como medida de asociación se utilizó la razón de prevalencia (RP) y su intervalo de 95% de confianza (IC95%), calculado mediante regresión de Poisson con varianza robusta, en modelos crudos y ajustados por covariables. La prevalencia de multimorbilidad fue del 52,1% (IC95%: 49,1-55,2), y fue significativamente mayor entre las mujeres (RP = 1,47; IC95% 1,29-1,67), con edades ≥ 40 años (40-59 años: RP = 1,49; IC95%: 1,28-1,73; ≥60 años: RP = 1,85; IC95%: 1,55-2,20) y menor para individuos con mayor nivel educativo (RP = 0,65; IC95%: 0,47-0,89). La prevalencia de multimorbilidad en la población indígena residente en aldeas en Espírito Santo fue mayor que la encontrada en otros estudios realizados en población general brasileña. Hubo asociación entre la presencia de multimorbilidad y el sexo, la edad y el nivel de escolaridad.

## Introduction

In recent decades, the Brazilian Indigenous population has undergone an intense process of epidemiological transition in health, in which noncommunicable diseases (NCDs) have emerged at an accelerated rate ^1^ ^,^ ^2^ ^,^ ^3^.

It is known that the increase in NCDs is associated with increased contact between Indigenous and non-Indigenous populations ^4^ ^,^ ^5^. This contact results from the expansion of agrarian borders, opening of new roads, environmental degradation, with limited access to food sources (hunting, fishing, fruit harvesting, etc.) leading to conflicts over the lands initially occupied by the Indigenous people. Such events significantly change the lifestyle of these peoples, especially change in dietary patterns, leading to increased consumption of alcohol, processed or ultra-processed foods, reduced physical activity, among others. All of these factors predispose to the development of various NCDs, including overweight/obesity, type II diabetes, arterial hypertension, and dyslipidemias ^1^. Furthermore, the contact of Indigenous populations with non-Indigenous populations, when associated with conflicts over ownership of the territory, increases psychosocial stress, which also predisposes to the onset of NCDs, such as hypertension and diabetes ^6^ ^,^ ^7^. This situation is aggravated by the traditionally more precarious access to health care services of peripheral communities, including Indigenous communities ^8^ ^,^ ^9^.

A meta-analysis by Kramer et al. ^10^ showed a 3.5 times higher prevalence of obesity among urbanized Indigenous people (28%) compared to those who lived on lands with more than 80% of native Amazon rainforest (8%). Other studies with Indigenous peoples have shown an increased prevalence of NCDs among them ^5^ ^,^ ^11^ ^,^ ^12^ ^,^ ^13^. These diseases do not seem to constitute isolated entities, as their onset depends both on specific genetic traits and also on factors related to lifestyle and the environment in which they live ^14^. This would be one of the reasons why such diseases can exist not only in isolation, but often coexist in the same individual, a situation that is known as multimorbidity ^15^.

Multimorbidity is widely addressed as the cooccurrence of two or more chronic diseases in the same individual ^15^. However, there is no consensus in the literature regarding its definition and the most appropriate methods for multimorbidity assessment, hampering studies comparison in this tophic ^16^. Therefore, this is an important global health issue ^17^ that mostly affects older adults ^18^, women ^19^ ^,^ ^20^, socioeconomically disadvantaged individuals, and residents of urban areas ^21^.

In recent years, interest in multimorbidity has increased, since this condition has been associated with several negative effects, such as increased mortality rate, decreased quality of life, excessive use of medical drugs (polypharmacy), higher demand for medical care, and, consequently, increased health care costs, as well as reduced productivity and functioning of individuals ^22^. These effects impact not only individuals affected by multimorbidity, but also their families, the health care system, especially within the scope of primary health care, and the society in which they live ^23^.

In Brazil, the health status of Indigenous peoples is still poorly known and to date there have been few studies on the prevalence of multimorbidity in this population ^24^ ^,^ ^25^ ^,^ ^26^. In other countries, studies describe a higher prevalence of multimorbidity among Indigenous people compared to non-Indigenous people ^27^ ^,^ ^28^. Moreover, it has been observed that, Indigenous people face the early onset of multimorbidity, compared to the non-Indigenous people ^29^.

Thus, given the complexity and challenge that multimorbidity represents for the health care system, especially in the context of primary health care, and the scarcity of studies on this condition in Brazilian Indigenous people, our objective was to determine the prevalence of multimorbidity and associated factors in the adult Indigenous population living in villages in the municipality of Aracruz, State of Espírito Santo, Brazil.

## Methods

This is a cross-sectional study using data from the research called *Assessment of the Prevalence and Severity of Chronic Diseases in the Indigenous Population of Espírito Santo State*. In Espírito Santos State, the population that self-declares as “Indigenous” is estimated at about 14,000 individuals. Of these, 4,663 (32.36%) reside in officially demarcated Indigenous lands (Comboios, Caieiras Velha II and Tupiniquim), located in the municipality of Aracruz, on the northern coast of Espírito Santo State, about 80km from the capital, Vitória. The population of these lands is divided into 12 villages. The Indigenous people are from the Tupiniquim ethnicity (about 80%) and Guarani ethnicity (about 10%), but there are also non-Indigenous residents in the villages ^30^ ^,^ ^31^. This population is served by five Indigenous primary health care units (UBSI, acronym in Portuguese): one located in a Guarani village (Boa Esperança) and the other in villages with a predominance of Tupiniquim population (Caieiras Velha, Irajá, Pau Brasil and Comboios). The study was publicized in the UBSI and in meetings with Indigenous leaders from all villages, and community health agents were responsible for invitations to participate.

Data collection began in September 2020 at a time when the COVID-19 pandemic was already declining in the Indigenous community of Aracruz. Even so, all collection followed the recommendations for preventing SARS-CoV-2 contamination ^32^. The collection was conducted daily in small groups of 3-4 participants, in a large space dedicated specifically to the project. The collection was extended until July 2022.

The invitation to participate in the study and the scheduling of examinations were conducted by community health agents, who were previously instructed on the nature and dynamics of the project, as well as on the need to travel to Vitória to perform the exams, which consisted in fasting blood collection (fasting for 12h to 14h) and clinical exams (anthropometry, blood pressure, bioimpedance, electrocardiogram, and pulse tonometry), in addition to data collection using questionnaires applied in interviews. Community health agents also communicated with the transportation provided by the project to pick up the participants on the scheduled day and time and transport them to the data collection site at the Cassiano Antônio Moraes University Hospital (HUCAM, acronym in Portuguese) at the Federal University of Espírito Santo (UFES, acronym in Portuguese). A team composed of interviewers and examiners received the participants to carry out the collection in a single morning. All team members were properly trained in accordance with the procedures adopted in the *Brazilian Longitudinal Study of Adult Health* (ELSA-Brasil, acronym in Portuguese) ^33^. The team was also trained on the adoption of protocols for prevention and protection against COVID-19.

To participate in the study, participants had to meet three inclusion criteria: be a resident of one of the villages in the reserve, be registered in one of the UBSI, and be aged ≥ 20 years. According to the registration of the Indigenous health coordination, there were 2,952 eligible persons (40.7% men) and of these 1,084 (36.7%) participated in the study. For this analysis, 108 participants who self-declared as non-Indigenous and 17 participants missing one or more outcome variables (14 dyslipidemia and three chronic kidney disease − CKD) were excluded, totaling 959 participants in the final sample.

The dependent variable was multimorbidity, defined by the presence of two or more chronic diseases in a group of eight morbidities (cancer, stroke, acute myocardial infarction, hypertension, diabetes, obesity, dyslipidemia, and CKD) with a prevalence ≥ 1% in the sample. In the total sum of morbidities, all had the same weight (equal to 1) ^15^.

The presence of cancer, stroke, and acute myocardial infarction was traced through the question “*Has any physician or other health care professional ever informed you that you have/had...?*” during the interview. The presence of other morbidities (obesity, hypertension, diabetes, dyslipidemia, and CKD) was traced by the results of the exams.

Blood pressure was measured in the left arm after resting for at least five minutes, in a silent setting with controlled temperature (20ºC to 24ºC), using a validated oscillometric device (Omron HEM 705CPINT; https://loja.omronbrasil.com/). Three measures were obtained at intervals of about one minute, and the mean of the last two measurements was considered ^33^. Arterial hypertension was defined by the presence of blood pressure ≥ 140/90mmHg or self-reported continuous use of antihypertensive medication, including diuretic agents ^34^.

Body weight was measured with individuals fasting, barefoot and without accessories, after emptying the bladder and wearing a standard uniform (400g-600g) on an electronic scale (Toledo; https://www.toledobrasil.com/) with a 0.1kg accuracy. Height was measured using a fixed wall stadiometer (Seca; https://www.seca.com/pt_mz.html) with a 0.1cm accuracy. Body mass index (BMI) was calculated as the ratio of weight (kg) to square height (m) and the value of ≥ 30kg/m² was used to trace individuals with obesity ^35^.

Diabetes was defined by the reported use of specific medication (oral hypoglycemic agents, including metformin, or insulin) and, when not reported, by the presence of fasting glycemia ≥ 126mg/dL, or glycemia ≥ 200mg/dL two hours after ingestion of a flavored solution of 75g of glucose (glucose tolerance test) or by the presence of glycated hemoglobin ≥ 6.5% ^36^.

Individuals who reported the use of lipid-lowering drugs (statins, fibrates or ezetimibe) and those who presented with altered values for LDL-c lipoprotein (≥ 160mg/dL), or triglycerides (≥ 150mg/dL) or HDL-c lipoprotein (< 40mg/dL in men or < 50mg/dL in women) were classified as dyslipidemic, according to the Update of the *Brazilian Directive on Dyslipidemia and Prevention of Atherosclerosis* ^37^.

Individuals with CKD were traced by self-reported participation in a chronic hemodialysis program or by presenting with a glomerular filtration rate (GFR) < 60mL/min/1.73m² calculated by the CKD-Epi formula, without correction for race/skin color ^38^.

The independent variables included sociodemographic variables collected by self-report in the interview: sex (male and female), age (20-39 years, 40-59 years, and ≥ 60 years), ethnicity (Tupiniquim, Guarani and other), and education level, indicating the highest level reached. Based on this information, subgroups (incomplete elementary education, incomplete secondary education, complete secondary education, and complete higher education) were defined. Furthermore, the variables smoking (never smoked, former smoker and current smoker) and alcohol consumption (never used, former user, user) were also included.

Descriptive statistics was used to synthesize the characteristics of the sample, calculating the prevalence for categorical variables and mean and standard deviation for continuous variables.

The differences in prevalence observed between individuals with and without multimorbidity according to independent variables were analyzed using the chi-square test. Also, crude and adjusted models were obtained using Poisson regression with robust variance to assess the association between the presence of multimorbidity and independent variables. Crude and adjusted prevalence ratios, their respective 95% confidence intervals (95%CI) and the p-value were obtained using this analysis. Data analysis used the SPSS version 22 (https://www.ibm.com/). Statistical significance was adopted for p < 0.05.

The research was approved by the Research Ethics Committee of the UFES Health Sciences Center and by the Brazilian National Research Ethics Committee (CONEP) (under the respective n. CAAE: 22563019.6.0000.5060, Opinion: 3.655.623; CONEP − CAAE: 22563019.6.0000.5060, Opinion: 3.828.655). All participants signed an informed consent form before data collection. Community health agents or researchers provided individuals with low literacy in Portuguese with specific help in reading and interpreting the form.

## Results

Of the sample analyzed, the majority (88.7%) were of the Tupiniquim ethnic group, followed by the Guarani ethnic group (9.9%) and a small group of other indigenous ethnic groups (1.2%). There was a higher proportion of women (57.6% vs. 42.4%) with a lower mean age than men (40.1±14.6 vs. 43.1±15.2 years; p < 0.002), most participants were aged from 20-39 years, had completed secondary education and reported never having used tobacco, while 15% reported using cigarettes currently and 40.6% being alcohol users (Table 1).

**Table 1:** Description of the sample according to demographic, socioeconomic and lifestyle variables of the Indigenous population living in villages. Aracruz, Espírito Santo State, Brazil (2020-2022).

Variables	n	%
Sex		
Male	407	42.4
Female	552	57.6
Age (years)		
20-39	487	50.8
40-59	334	34.8
≥ 60	138	14.4
Ethnicity		
Tupiniquim	851	88.7
Guarani	95	9.9
Other	13	1.4
Education [n = 956]		
Incomplete elementary education	304	31.7
Incomplete secondary education	200	20.9
Complete secondary education	357	37.2
Incomplete or complete higher education	95	9.9
Smoking		
Never smoked	605	63.1
Former smoker	210	21.9
Current smoker	144	15.0
Alcohol consumption [n = 952]		
Never used	305	31.8
Former user	258	26.9
User	389	40.6

A mean of 1.7±1.2 morbidities/subject was observed in the total sample. Table 2 shows the prevalence of morbidities in the sample for the Tupiniquim and Guarani ethnic groups. It was observed that dyslipidemia (72.7%), obesity (37.5) and hypertension (34.9%) were the most prevalent conditions, while CKD (1.3%), stroke (1.4%) and cancer (1%) presented lower frequencies. The morbidities that presented statistically significant differences between the ethnicities were obesity and hypertension (38.9% vs 25.3% p = 0.009; 37% vs 15.8% p < 0.001, respectively) with the lowest values in the Guarani people.

**Table 2:** Prevalence of morbidities in the adult Indigenous population living in villages, according to ethnicity. Aracruz, Espírito Santo State, Brazil (2020-2022).

Morbidity	Total [n = 946]	Tupiniquim [n = 851]	Guarani [n = 95]	p-value
	n (%)	n (%)	n (%)	
Dyslipidemia	688 (72.7)	612 (71.9)	76 (80.0)	0.093
Obesity	355 (37.5)	331 (38.9)	24 (25.3)	0.009
Arterial hypertension	330 (34.9)	315 (37.0)	15 (15.8)	< 0.001
Diabetes mellitus	187 (19.8)	174 (20.4)	13 (13.7)	0.116
Acute myocardial infarction	12 (1.3)	11 (1.3)	1 (1.0)	0.843
Chronic kidney disease	13 (1.4)	13 (1.5)	0 (0.0)	*
Cerebral vascular accident	13 (1.4)	13 (1.5)	0 (0.0)	*
Cancer	10 (1.0)	10 (1.2)	0 (0.0)	*

Note: chi-square test.

* No statistical test was applied.

The overall prevalence of multimorbidity in the sample was 52.1% (95%CI: 49.1-55.2), being significantly higher among women, in individuals who were 60 years or older, of the Tupiniquim ethnic group, with incomplete elementary education, former smokers and former alcohol users (Table 3).

**Table 3:** Prevalence of multimorbidity according to independent variables in the adult Indigenous population living in villages. Aracruz, Espírito Santo State, Brazil (2020-2022).

Variables	Total (n)	Multimorbidity (≥ 2 morbidities)		p-value
		No n (%)	Yes n (%)	
Sex				< 0.001
Male	407	231 (56.8)	176 (43.2)	
Female	552	228 (41.3)	324 (58.7)	
Age (years)				< 0.001
20-39	487	299 (61.4)	188 (38.6)	
40-59	334	131 (39.2)	203 (60.8)	
≥ 60	138	29 (21.0)	109 (79.0)	
Ethnicity *				0.013
Tupiniquim	851	396 (46.5)	455 (53.6)	
Guarani	95	57 (60.0)	38 (40.0)	
Education [n = 956]				< 0.001
Incomplete elementary education	304	112 (36.8)	192 (63.2)	
Incomplete secondary education	200	89 (44.5)	111 (55.5)	
Complete secondary education	357	190 (53.2)	167 (46.8)	
Incomplete or complete higher education	95	65 (68.4)	30 (31.6)	
Smoking				< 0.001
Never smoked	605	305 (50.4)	300 (49.6)	
Former smoker	210	74 (35.2)	136 (64.8)	
Current smoker	144	80 (55.6)	64 (44.4)	
Alcohol consumption [n = 952]				0.015
Never used	305	139 (45.6)	166 (54.4)	
Former user	258	109 (42.2)	149 (57.8)	
User	389	207 (53.2)	182 (46.8)	

* For this analysis, individuals who self-declared to be of another ethnicity were not included due to the small n in the sample.

When analyzing the prevalence ratio, it was found that the probability of presence of multimorbidity was higher in women, aged ≥ 40 years and lower for individuals with higher education (Table 4).

**Table 4:** Multimorbidity prevalence ratio (PR) according to independent variables in the adult Indigenous population living in villages. Aracruz, Espírito Santo State, Brazil (2020-2022).

Variables	Crude analysis			Adjusted analysis *		
	PR	95%CI	p-value	PR	95%CI	p-value
Sex						
Male	1.00			1.00		
Female	1.36	1.19-1.55	< 0.001	1.50	1.32-1.70	< 0.001
Age (years)						
20-39	1.00			1.00		
40-59	1.55	1.35-1.79	< 0.001	1.50	1.29-1.74	< 0.001
≥ 60	2.04	1.76- 2.35	< 0.001	1.82	1.52-2.17	< 0.001
Ethnicity **						
Tupiniquim	1.00			1.00		
Guarani	0.74	0.57-0.96	0.023	0.78	0.60-1.03	0.078
Education						
Incomplete elementary education	1.00			1.00		
Incomplete secondary education	0.88	0.76-1.02	0.097	1.05	0.91-1.21	0.507
Complete secondary education	0.74	0.64-0.85	< 0.001	0.96	0.81-1.14	0.616
Incomplete or complete higher education	0.51	0.37-0.69	< 0.001	0.63	0.46-0.87	0.005
Smoking						
Never smoked	1.00			1.00		
Former smoker	1.29	1.14-1.47	< 0.001	1.13	0.98-1.30	0.082
Current smoker	0.89	0.73-1.09	0.246	0.97	0.79-1.20	0.800
Alcohol consumption						
Never used	1.00			1.00		
Former user	1.06	0.92-1.23	0.422	1.06	0.91-1.23	0.432
User	0.86	0.74-0.99	0.043	0.97	0.83-1.13	0.689

95%CI: 95% confidence interval.

* Adjustment performed for all independent variables in the table;

** For this analysis, individuals who self-declared to be of another ethnicity were not included due to the small n in the sample.

## Discussion

This study analyzes the prevalence of multimorbidity in the Indigenous population living in villages in the municipality of Aracruz. The findings show a high prevalence of multimorbidity among the whole sample. In addition, the presence of multimorbidity was more frequent in women, older individuals and those with lower education.

According to data from the *Brazilian National Health Survey* (PNS, acronym in Portuguese), the prevalence of multimorbidity in the Brazilian adult population increased from 18.7% (95%CI: 18.0-19.3) in 2013 to 22.3% (95%CI: 21.7-22.9) in 2019 ^39^. Other studies conducted in various locations in Brazil presented estimates ranging from 10.9% to 41.5% ^40^ ^,^ ^41^ ^,^ ^42^ ^,^ ^43^ ^,^ ^44^. Therefore, this study shows that the prevalence found (51.4%) is higher than that observed in the general Brazilian population ^39^ and in specific populations.

The finding of this study is consistent with studies in native populations from other countries, such as Australia and Canada. Randall et al. ^28^, when comparing Indigenous people with non-indigenous people in Australia, found a higher probability of multimorbidity among indigenous people (2.59; 95%CI: 2.55-2.62) when compared to non-Indigenous people. Also in Australia, there was a higher prevalence of multimorbidity among aborigines (24.2%) compared to non-aborigines (20.7%) ^45^. In Canada, Kuwornu et al. ^46^ also found a higher prevalence of multimorbidity among the Indigenous group (38.9%; 95%CI: 36.5-41.3) when compared to non-Indigenous people (30.7%; 95%CI: 28.9-32.6).

In Brazil, studies assessing the prevalence of multimorbidity specifically in the Indigenous population have not been found as of the time of this research. The *First National Survey of Indigenous People’s Health and Nutrition* ^9^, conducted in 2009, was an important milestone for the overall health of Brazilian Indigenous peoples. However, most of the data were collected from indigenous groups in the Amazon and included only women and children. The work enabled understanding the actual health situation of women and children, indicating the emergence of NCDs among women ^9^.

Our study found that two morbidities − obesity and diabetes − were very frequent, with prevalences of 37.5% and 19.8%, respectively, which are higher than the corresponding prevalences of 25.9% and 7.7% found in the general Brazilian population studied in the 2019 PNS ^47^ ^,^ ^48^. The increased prevalence of these morbidities in Indigenous peoples in Brazil has been reported in several studies ^11^ ^,^ ^49^ ^,^ ^50^ ^,^ ^51^ ^,^ ^52^ ^,^ ^53^ and the rapid growth of this condition in Indigenous people indicates an increase in the incidence of type 2 diabetes, which, apparently, is already observed in the population of this study. However, it should be noted that the cross-sectional characteristic of the data allows no causal inference between these conditions.

Notably, when addressing indigenous peoples in Brazil, it should be considered that there are populations residing in isolated reserves and populations living in urban areas or surrounding urban areas ^54^. The Indigenous population of Aracruz lives on land that has already been demarcated, but they keep in strong contact with the rural and urban populations around the reserve. This contact enables these Indigenous people to have easy access to means of transportation − since bus lines serve the villages −, food purchased in supermarkets, and communication technologies, among others. In this context, studies have shown the extent to which the proximity of Indigenous territories to the urban setting has contributed to the increased incidence of NCDs among Indigenous people ^10^ ^,^ ^11^.

When analyzing the prevalence of morbidities for the Tupiniquim and Guarani ethnic groups, a significant difference was found in the prevalence of obesity and hypertension. However, when multimorbidity is analyzed, the difference between ethnicities remains significant only in the crude analyses. This finding can be attributed to the fact that the difference was found only in the morbidities that presented higher prevalence in the sample (obesity and hypertension).

Among the factors associated with the outcome, women were more likely to present multimorbidity when compared to men. Similar results were found in other studies conducted in Brazil ^55^ ^,^ ^56^ ^,^ ^57^. This finding can be explained by the fact that women in this study have a higher prevalence of obesity compared to men (46.5% vs. 25.2%; p < 0.05 - data not shown), a morbidity that has a strong association with type II diabetes and hypertension. In fact, as women use health care services more often, are more likely to receive a medical diagnosis ^58^. This does not seem to be the case, because obesity, hypertension, diabetes and dyslipidemia were defined not by self-report, but by tests. The morbidities reported were much less frequent. Furthermore, studies have associated the higher prevalence of obesity in Indigenous women with the fact that they mostly perform handicraft activities, which require less energy expenditure when compared to the work activities of Indigenous men, who mostly work in the crop ^49^ ^,^ ^59^ ^,^ ^60^. Survival bias, since men have lower life expectancy, could also explain this difference, but this does not seem to be the case, since the distribution of ages was similar between men and women, with a slightly lower mean in women ^63^.

As expected, there was an increase in the prevalence of multimorbidity with aging. A systematic review by Violan et al. ^62^ showed that age was the determinant that showed positive and significant association with multimorbidity in all studies reviewed. The same authors explain that this association is due to older individuals being more exposed to stressors throughout life, which compromises physiological balance, favoring the development of chronic diseases.

Another factor associated with multimorbidity was education. Although, in the adjusted analysis, the education variable lost part of its explanatory capacity, this reduction can be attributed to the presence of the age variable. This is due to the outcome studied being predominant in older individuals, who generally have a lower education level ^63^. Education level is an important factor for this outcome, since it enables individuals to seek knowledge about health promotion, as well as the adoption of healthier lifestyles and consequently the prevention of chronic diseases ^64^.

The results found in this study should be interpreted in the context of its limitations. These limitations include the sample nor being representative of the Indigenous population living in villages in Aracruz, as the study was carried out on volunteers, which hinders extrapolation of the data presented to the Indigenous population in general. However, the data presented most likely reflect the actual situation of the Tupiniquins and Guaranis living in the reserve where the study was carried out. One of the difficulties to reach a more robust sample was due to the study period − during the COVID-19 pandemic − which led some persons to give no response to the research call. A small return bias occurred regarding sex, as in the villages the male population is 49.3% and in the sample it was 42.4%. Regarding age groups, the sample is slightly older than the general population, in which the frequency of individuals aged 20-39 years is 58.9% and in the sample it was 50%, while the older adults (≥ 60 years) represent 12.4% of the general population and 14.4% in the studied sample. Even so, it can be inferred that the general situation of morbidities in the population is very similar to that described in the sample. Furthermore, the need to travel to Vitória to undergo the exams may have hindered the participation of people with limitations to be absent from work.

Another limitation is the differences between the aspects of measuring multimorbidity, which makes it difficult to compare studies. Moreover, these study employed only eight chronic morbidities to trace multimorbidity, whereas studies recommend the use of at least 12 morbidities ^65^. However, it is noted that this study prioritized the use of morbidities that, when not previously diagnosed, could be measured in the study itself, thus favoring the strength of the associations found.

Finally, there are the difficulties inherent to the cross-sectional study, which does not allow us to infer associations of cause and effect, which may have impaired the assessment of some associations. On the other hand, the value of this study lies in the novelty of presenting the prevalence of multimorbidity in an Indigenous population of Brazil that lives in villages with a strong relationship with the urban setting − as occurs with many other Indigenous populations nationwide.

The findings on multimorbidity in the indigenous population provide an important foundation for future analyses of the adoption of measures to prevent NCDs, of the supply and sustainability of the primary health care service offered in this population. The results of this research show a high prevalence of multimorbidity in this population, associated with women, age and education level, demonstrating that it is necessary to prioritize prevention strategies in these groups.
