# Supplementary material for: Multimorbidity and associated factors in the adult Indigenous population living in villages in the municipality of Aracruz, Espírito Santo, State, Brazil
Source: Cad Saude Publica. 2025 Feb 7;40(12):e00135323. doi: 10.1590/0102-311XEN135323 (PMC11805521; doi:10.1590/0102-311XEN135323)
Supplement: Translation

# Translation: Multimorbidade e fatores associados na população indígena adulta aldeada no Município de Aracruz, Espírito Santo, Brasil

**DOI:** 10.1590/0102-311XPT135323

**Authors:** Hully Cantão dos Santos, José Geraldo Mill

**Affiliations:** 1 Universidade Federal do Espírito Santo, Vitória, Brasil.

**Keywords:** Saúde de Populações Indígenas, Multimorbidade, Doença Crônica, Prevalência

## Abstract

A multimorbidade está associada a efeitos negativos sobre a saúde dos indivíduos, aumentando a complexidade da assistência à saúde. Nosso objetivo foi determinar a prevalência de multimorbidade e fatores associados na população indígena adulta aldeada em Aracruz, Espírito Santo, Brasil. Trata-se de estudo transversal realizado com os dados do projeto *Avaliação da Prevalência e Severidade das Doenças Crônicas na População Indígena do Espírito Santos*. A coleta de dados foi realizada entre 2020 e 2022. A multimorbidade foi definida pela presença de duas ou mais morbidades crônicas em um grupo de oito morbidades. Como medida de associação, utilizou-se a razão de prevalência (RP) e seu intervalo de 95% de confiança (IC95%), calculados por regressão de Poisson com variância robusta, em modelos bruto e ajustado por covariáveis. A prevalência de multimorbidade foi de 52,1% (IC95%: 49,1-55,2) sendo significativamente maior entre as mulheres (RP = 1,47; IC95%: 1,29-1,67), idade ≥ 40 anos (40-59 anos: RP = 1,49; IC95%: 1,28-1,73; ≥ 60 anos: RP = 1,85; IC95%: 1,55-2,20) e menor para os indivíduos com nível superior de escolaridade (RP = 0,65; IC95%: 0,47-0,89). A prevalência de multimorbidade na população indígena aldeada do ES foi superior à encontrada em outros estudos realizados na população geral brasileira. Houve associação da presença de multimorbidade com sexo, idade e nível de escolaridade.

## Introdução

Nas últimas décadas, a população indígena brasileira tem passado por um intenso processo de transição epidemiológica em saúde, na qual a emergência das doenças crônicas não transmissíveis (DCNT) tem ocorrido de forma acelerada ^1^ ^,^ ^2^ ^,^ ^3^.

Sabe-se que o aumento das DCNT está associado com o aumento do contato da população indígena com a não indígena ^4^ ^,^ ^5^. Esse contato decorre da expansão das fronteiras agrárias, abertura de novas estradas, degradação ambiental, com limitação de acesso a itens de alimentação (caça, pesca, frutos etc.), levando a conflitos pelas terras inicialmente ocupadas pelos indígenas. Tais acontecimentos provocam mudanças significativas no estilo de vida desses povos, com destaque para a alteração no padrão alimentar, levando ao aumento de consumo de álcool, de alimentos processados ou ultraprocessados, à redução de atividade física, entre outros. Todos esses fatores predispõem ao desenvolvimento de várias DCNT, incluindo sobrepeso/obesidade, diabetes do tipo II, hipertensão arterial e dislipidemias ^1^. Além disso, o contato das populações indígenas com as não indígenas, quando associado aos conflitos pela posse do território, aumenta o estresse psicossocial, o que também predispõe ao aparecimento de DCNT, como hipertensão e diabetes ^6^ ^,^ ^7^. Esse quadro se agrava porque, tradicionalmente, o acesso aos serviços de saúde é mais precário para as comunidades periféricas, incluindo as comunidades indígenas ^8^ ^,^ ^9^.

Metanálise realizada por Kramer et al. ^10^ mostrou prevalência 3,5 vezes maior de obesidade entre indígenas urbanizados (28%) em comparação com aqueles que viviam em terras com mais de 80% de floresta amazônica nativa (8%). Outros estudos realizados com povos indígenas têm mostrado um aumento da prevalência de DCNT entre eles ^5^ ^,^ ^11^ ^,^ ^12^ ^,^ ^13^. Essas doenças não parecem se constituir em entidades isoladas, pois seu aparecimento depende tanto de traços genéticos específicos como de fatores ligados ao estilo de vida e ao ambiente ^14^. Essa seria uma das razões pelas quais tais doenças podem se apresentar não só de forma isolada, mas frequentemente coexistirem em um mesmo indivíduo, situação conhecida como multimorbidade ^15^.

A multimorbidade é amplamente abordada como a coocorrência de duas ou mais doenças crônicas no mesmo indivíduo ^15^. No entanto, não há um consenso na literatura quanto à sua definição e aos métodos mais apropriados para a sua avaliação, dificultando as comparações entre os estudos nessa área ^16^. Trata-se, portanto, de um importante problema de saúde global ^17^ que acomete principalmente idosos ^18^, mulheres ^19^ ^,^ ^20^, indivíduos socioeconomicamente desfavorecidos e moradores de áreas urbanas ^21^.

Nos últimos anos, o interesse por essa condição vem crescendo, uma vez que é associada a diversos efeitos negativos, tais como o aumento da taxa de mortalidade, a diminuição da qualidade de vida, o uso excessivo de medicamentos (polifarmácia), maior demanda por assistência médica e, consequentemente, aumento dos custos com a saúde, bem como redução da produtividade e da capacidade funcional dos indivíduos ^22^. Esses efeitos impactam não só o indivíduo acometido pela multimorbidade, como também suas famílias, o sistema de saúde, principalmente no âmbito da atenção primária, e a sociedade na qual está inserido ^23^.

No Brasil, o estado de saúde dos povos indígenas ainda é pouco conhecido e, até o momento, há poucos estudos de avaliação da prevalência de multimorbidade neste segmento da população brasileira ^24^ ^,^ ^25^ ^,^ ^26^. Em outros países, os estudos descrevem maior prevalência de multimorbidade entre indígenas em comparação com não indígenas ^27^ ^,^ ^28^. Além disso, tem-se observado que, em indígenas, a multimorbidade se manifesta em idade mais precoce do que em não indígenas ^29^.

Com isso, diante da complexidade e do desafio que a multimorbidade representa para o sistema de saúde, principalmente no âmbito da atenção primária e da escassez de estudos que avaliam tal condição em indígenas brasileiros, nosso objetivo foi determinar a prevalência de multimorbidade e fatores associados na população indígena adulta aldeada em Aracruz, no Espírito Santo, Brasil.

## Métodos

Estudo transversal realizado com dados da pesquisa *Avaliação da Prevalência e Severidade das Doenças Crônicas na População Indígena do Espírito Santo*. No Espírito Santo a população que se autodeclara indígena é estimada em cerca de 14 mil indivíduos. Desses, 4.663 (32,36%) residem nas terras indígenas oficialmente demarcadas (Comboios, Caieiras Velha II e Tupiniquim), localizadas no Município de Aracruz, no litoral norte do Espírito Santo, cerca de 80km distante da capital, Vitória. A população dessas terras está dividida em 12 aldeias. Os indígenas são das etnias Tupiniquim (cerca de 80%) e Guarani (cerca de 10%), mas há também moradores não indígenas nas aldeias ^30^ ^,^ ^31^. A atenção primária à saúde dessa população é fornecida por cinco unidades básicas de saúde indígena (UBSI), sendo uma localizada em aldeia Guarani (Boa Esperança) e as demais em aldeias com predominância de população Tupiniquim (Caieiras Velha, Irajá, Pau Brasil e Comboios). A divulgação do estudo foi feito nas UBSI e em reuniões com as lideranças indígenas de todas as aldeias, cabendo aos agentes comunitários de saúde realizarem os convites para participação.

A coleta de dados foi iniciada em setembro de 2020, no momento em que a pandemia de COVID-19 apresentava declínio na comunidade indígena de Aracruz. Mesmo assim, toda a coleta foi realizada seguindo as recomendações de prevenção de contaminação pelo SARS-CoV-2 ^32^. O atendimento era feito diariamente, em grupos de 3-4 participantes, em espaço amplo e dedicado especificamente ao projeto. A coleta se estendeu até julho de 2022.

O convite para a participação no estudo e o dia de agendamento dos exames foi feito pelos agentes comunitários de saúde previamente instruídos sobre a natureza e a dinâmica do projeto, bem como a necessidade do deslocamento até Vitória para a realização dos exames, que consistiam em coleta de sangue em jejum (jejum de 12 a 14 horas) e exames clínicos (antropometria, pressão arterial, bioimpedância, eletrocardiograma e tonometria de pulso), além da coleta de dados por questionários aplicados em entrevistas. Os agentes comunitários de saúde também faziam a comunicação com o transporte disponibilizado pelo projeto para buscar os participantes no dia e horário marcados e transportá-los até o local da coleta de dados no Hospital Universitário Cassiano Antônio Moraes (HUCAM) na Universidade Federal do Espírito Santo (UFES). Uma equipe composta por entrevistadores e aferidores recebia os participantes para fazer a coleta numa única manhã. Todos os integrantes da equipe foram devidamente treinados de acordo com os procedimentos adotados no projeto *Estudo Longitudinal de Saúde do Adulto* (ELSA-Brasil) ^33^. A equipe também foi treinada quanto à adoção de protocolos de prevenção e proteção contra a COVID-19.

Para participar do estudo, o participante deveria atender a três critérios de inclusão: ser morador de uma das aldeias da reserva; ter cadastro em uma das UBSI; e ter idade ≥ 20 anos. Segundo cadastro da coordenação de saúde indígena, havia 2.952 elegíveis (40,7% de homens), e, desses, 1.084 (36.7%) participaram do estudo. Para esta análise, foram excluídos 108 participantes que se declararam não indígenas e 17 participantes com falta de uma ou mais variáveis de desfecho (14 dislipidemia e três doença renal crônica − DRC), resultando em uma amostra final de 959 participantes.

A variável dependente foi a multimorbidade, definida pela presença de duas ou mais doenças crônicas em um grupo de oito morbidades (câncer, acidente vascular cerebral - AVC, infarto agudo do miocárdio, hipertensão arterial, diabetes, obesidade, dislipidemia e DRC) com prevalência ≥ 1% na amostra. No somatório total de morbidades, todas tiveram o mesmo peso (igual a 1) ^15^.

A presença de câncer, AVC e infarto agudo do miocárdio foi identificada por meio da pergunta “*algum médico ou outro profissional de saúde já informou que o senhor(a) tem/teve…?*” durante entrevista. A presença das demais morbidades (obesidade, hipertensão arterial, diabetes, dislipidemia e DRC foi identificada pelo resultado dos exames.

A pressão arterial foi medida no braço esquerdo após repouso de pelo menos cinco minutos, em ambiente silencioso com temperatura controlada (20 a 24ºC), utilizando um aparelho oscilométrico validado (Omron HEM 705CPINT; https://loja.omronbrasil.com/). Foram obtidas três medidas em intervalos de cerca de um minuto, sendo considerada a média das duas últimas medidas ^33^. A hipertensão arterial foi definida pela presença de pressão arterial ≥ 140/90mmHg ou autorrelato de uso contínuo de medicamento anti-hipertensivo, incluindo diurético ^34^.

O peso corporal foi aferido com o indivíduo em jejum, descalço e sem adereços, após esvaziamento vesical e usando uniforme padrão (400-600g) em balança eletrônica (Toledo; https://www.toledobrasil.com/) com precisão de 0,1kg. A estatura foi aferida em estadiômetro fixo de parede (Seca; https://www.seca.com/pt_mz.html) com precisão de 0,1cm. O índice de massa corporal (IMC) foi calculado por meio da razão do peso (kg) pelo quadrado da estatura (m²), e o valor ≥ 30kg/m² foi utilizado para identificar os indivíduos com obesidade ^35^.

O diabetes foi definido por meio do relato de uso de medicação específica (hipoglicemiantes orais, incluindo metformina, ou insulina) e, quando não relatado, pela presença de glicemia de jejum ≥ 126mg/dL, ou glicemia ≥ 200mg/dL duas horas após ingestão de solução flavorizada de 75g de glicose (teste de tolerância à glicose) ou pela presença de hemoglobina glicada ≥ 6,5% ^36^.

Foram classificados com dislipidemia os indivíduos que relataram uso de hipolipemiantes (estatinas, fibratos ou ezetimiba) e aqueles que apresentaram alteração nos valores da lipoproteína LDL-c (≥ 160mg/dL) ou triglicerídeos (≥ 150mg/dL) ou na lipoproteína HDL-c (< 40mg/dL em homens ou < 50mg/dL em mulheres), conforme a *Atualização da Diretriz Brasileira de Dislipidemia e Prevenção da Aterosclerose* ^37^.

Os indivíduos com DRC foram identificados a partir do autorrelato de estarem em programa de hemodiálise crônica ou com taxa de filtração glomerular (TFG) < 60mL/min/1,73m² calculada pela fórmula CKD-Epi, sem correção para raça/cor ^38^.

As variáveis independentes incluíram variáveis sociodemográficas coletadas por autorrelato em entrevista: sexo (masculino e feminino), idade (20-39 anos, 40-59 anos e ≥ 60 anos), etnia (Tupiniquim, Guarani e outra) e escolaridade, indicando o maior nível atingido. A partir dessa informação, foram constituídos os subgrupos (Ensino Fundamental incompleto, Ensino Médio incompleto, Ensino Médio completo e Ensino Superior). Além dessas, também foi incluída a variável de tabagismo (nunca fumou, ex-fumante e fuma atualmente) e consumo de bebida alcoólica (nunca usou, ex-usuário, usuário).

A estatística descritiva foi utilizada para sintetizar as características da amostra, calculando-se a prevalência para as variáveis categóricas e a média e o desvio padrão para as variáveis contínuas.

As diferenças nas prevalências observadas entre os indivíduos com e sem multimorbidade segundo as variáveis independentes foram analisadas por meio do teste de qui-quadrado. Além disso, modelos bruto e ajustado foram obtidos por meio da regressão de Poisson com variância robusta para avaliar a associação entre a presença de multimorbidade e as variáveis independentes. Por meio dessa análise, foram obtidas as razões de prevalência bruta e ajustada, seus respectivos intervalos de 95% de confiança (IC95%) e o valor de p. A análise dos dados foi realizada com o softwere SPSS, versão 22 (https://www.ibm.com/). A significância estatística foi adotada para p < 0,05.

A pesquisa foi aprovada pelo Comitê de Ética e Pesquisa (CEP) do Centro de Ciências da Saúde da UFES e pela Comissão Nacional de Ética e Pesquisa (Conep) (sob os respectivos registros: CEP - CAAE: 22563019.6.0000.5060, parecer nº 3.655.623; Conep - CAAE: 22563019.6.0000.5060, Parecer nº 3.828.655). Todos os participantes assinaram o Termo de Consentimento Livre e Esclarecido (TCLE) antes da coleta de dados. Foi dada ajuda específica na leitura e na interpretação do TCLE pelas agentes comunitárias de saúde ou pelos pesquisadores aos indivíduos com baixo letramento na língua portuguesa.

## Resultados

Da amostra analisada, a maioria (88,7%) era da etnia Tupiniquim, seguido da etnia Guarani (9,9%) e pequeno grupo de outras etnias indígenas (1,2%). Observou-se maior proporção de mulheres (57,6% *vs.* 42,4%) com média de idade menor do que em homens (40,1±14,6 *vs.* 43,1±15,2 anos; p < 0,002), a maioria dos participantes tinha de 20-39 anos, apresentava Ensino Médio completo e relatou nunca ter feito uso de tabaco, enquanto 15% relataram fazer uso de cigarro atualmente e 40,6% serem usuários de bebida alcoólica (Tabela 1).

**Tabela 1:** Descrição da amostra segundo variáveis demográficas, socioeconômicas e hábitos de vida da população indígena aldeada. Município de Aracruz, Espírito Santo, Brasil (2020-2022).

Variável	n	%
Sexo		
Masculino	407	42,4
Feminino	552	57,6
Idade (anos)		
20-39	487	50,8
40-59	334	34,8
≥ 60	138	14,4
Etnia		
Tupiniquim	851	88,7
Guarani	95	9,9
Outra	13	1,4
Escolaridade [n = 956]		
Ensino Fundamental incompleto	304	31,7
Ensino Médio incompleto	200	20,9
Ensino Médio completo	357	37,2
Ensino Superior (incompleto ou completo)	95	9,9
Tabagismo		
Nunca fumou	605	63,1
Ex-fumante	210	21,9
Fuma atualmente	144	15,0
Consumo de álcool [n = 952]		
Nunca usou	305	31,8
Ex-usuário	258	26,9
Usuário	389	40,6

Observou-se uma média de 1,7±1,2 morbidades/indivíduo na amostra total. As prevalências das morbidades na amostra de acordo com as etnias Tupiniquim e Guarani são apresentadas na Tabela 2. Observa-se que dislipidemia (72,7%), obesidade (37,5) e hipertensão arterial (34,9%) foram as mais prevalentes, enquanto DRC (1,3%), AVC (1,4%) e câncer (1%) apresentaram frequências menores. As morbidades que apresentaram diferenças estatisticamente significativas entre as etnias foram obesidade e hipertensão arterial (38,9% *vs.* 25,3% p = 0,009; 37% *vs.* 15,8% p < 0,001, respectivamente) com os menores valores na etnia Guarani.

**Tabela 2:** Prevalência de morbidades na população indígena adulta aldeada, segundo etnia. Município de Aracruz, Espírito Santo, Brasil (2020-2022).

Morbidades	Total [n = 946]	Tupiniquim [n = 851]	Guarani [n = 95]	Valor de p
	n (%)	n (%)	n (%)	
Dislipidemia	688 (72,7)	612 (71,9)	76 (80,0)	0,093
Obesidade	355 (37,5)	331 (38,9)	24 (25,3)	0,009
Hipertensão arterial	330 (34,9)	315 (37,0)	15 (15,8)	< 0,001
Diabetes mellitus	187 (19,8)	174 (20,4)	13 (13,7)	0,116
Infarto agudo do miocárdio	12 (1,3)	11 (1,3)	1 (1,0)	0,843
Doença renal crônica	13 (1,4)	13 (1,5)	0 (0,0)	*
Acidente vascular cerebral	13 (1,4)	13 (1,5)	0 (0,0)	*
Câncer	10 (1,0)	10 (1,2)	0 (0,0)	*

Nota: teste qui-quadrado.

* Não foi aplicado teste estatístico.

A prevalência geral de multimorbidade na amostra foi de 52,1% (IC95%: 49,1-55,2), sendo significativamente maior entre as mulheres, nos indivíduos que apresentavam 60 anos ou mais, da etnia Tupiniquim, Ensino Fundamental incompleto, eram ex-fumantes e ex-usuários de álcool (Tabela 3).

**Tabela 3:** Prevalência de multimorbidade segundo variáveis independentes na população indígena adulta aldeada. Município de Aracruz, Espírito Santo, Brasil (2020-2022).

Variáveis	Total	Multimorbidade (≥ 2 morbidades)		Valor de p
		Não n (%)	Sim n (%)	
Sexo				< 0,001
Masculino	407	231 (56,8)	176 (43,2)	
Feminino	552	228 (41,3)	324 (58,7)	
Idade (anos)				< 0,001
20-39	487	299 (61,4)	188 (38,6)	
40-59	334	131 (39,2)	203 (60,8)	
≥ 60	138	29 (21,0)	109 (79,0)	
Etnia *				0,013
Tupiniquim	851	396 (46,5)	455 (53,6)	
Guarani	95	57 (60,0)	38 (40,0)	
Escolaridade [n = 956]				< 0,001
Ensino Fundamental incompleto	304	112 (36,8)	192 (63,2)	
Ensino Médio incompleto	200	89 (44,5)	111 (55,5)	
Ensino Médio completo	357	190 (53,2)	167 (46,8)	
Ensino Superior (incompleto ou completo)	95	65 (68,4)	30 (31,6)	
Tabagismo				< 0,001
Nunca fumou	605	305 (50,4)	300 (49,6)	
Ex-fumante	210	74 (35,2)	136 (64,8)	
Fuma atualmente	144	80 (55,6)	64 (44,4)	
Consumo de álcool [n = 952]				0,015
Nunca usou	305	139 (45,6)	166 (54,4)	
Ex-usuário	258	109 (42,2)	149 (57,8)	
Usuário	389	207 (53,2)	182 (46,8)	

* Para essa análise, não foram incluídos os indivíduos que se autodeclararam de outra etnia devido ao n pequeno na amostra.

Ao analisarmos a razão de prevalência, verificou-se que a probabilidade de presença de multimorbidade foi maior nos indivíduos do sexo feminino, com idade ≥ 40 anos e menor para os indivíduos com nível superior de escolaridade (Tabela 4).

**Tabela 4:** Razão de prevalência de multimorbidade segundo variáveis independentes na população indígena adulta aldeada. Município de Aracruz, Espírito Santo, Brasil (2020-2022).

Variáveis	Análise bruta			Análise ajustada *		
	RP	IC95%	Valor de p	RP	IC95%	Valor de p
Sexo						
Masculino	1,00			1,00		
Feminino	1,36	1,19-1,55	< 0,001	1,50	1,32-1,70	< 0,001
Idade (anos)						
20-39	1,00			1,00		
40-59	1,55	1,35-1,79	< 0,001	1,50	1,29-1,74	< 0,001
≥ 60	2,04	1,76-2,35	< 0,001	1,82	1,52-2,17	< 0,001
Etnia **						
Tupiniquim	1,00			1,00		
Guarani	0,74	0,57-0,96	0,023	0,78	0,60-1,03	0,078
Escolaridade						
Ensino Fundamental incompleto	1,00			1,00		
Ensino Médio incompleto	0,88	0,76-1,02	0,097	1,05	0,91-1,21	0,507
Ensino Médio completo	0,74	0,64-0,85	< 0,001	0,96	0,81-1,14	0,616
Ensino Superior (incompleto ou completo)	0,51	0,37-0,69	< 0,001	0,63	0,46-0,87	0,005
Tabagismo						
Nunca fumou	1,00			1,00		
Ex-fumante	1,29	1,14-1,47	< 0,001	1,13	0,98-1,30	0,082
Fuma atualmente	0,89	0,73-1,09	0,246	0,97	0,79-1,20	0,800
Consumo de álcool						
Nunca usou	1,00			1,00		
Ex-usuário	1,06	0,92-1,23	0,422	1,06	0,91-1,23	0,432
Usuário	0,86	0,74-0,99	0,043	0,97	0,83-1,13	0,689

IC95%: intervalo de 95% de confiança; RP: razão de prevalência.

* Ajuste realizado para todas as variáveis independentes da tabela;

** Para essa análise, não foram incluídos os indivíduos que se autodeclararam de outra etnia devido ao n pequeno na amostra.

## Discussão

Este estudo analisa a prevalência de multimorbidade na população indígena aldeada no Município de Aracruz. Os achados evidenciam uma alta prevalência de multimorbidade em toda a amostra. Além disso, a presença de multimorbidade foi mais frequente em mulheres, nos indivíduos mais velhos e naqueles com escolaridade mais baixa.

Segundo dados da *Pesquisa Nacional de Saúde* (PNS), a prevalência de multimorbidade na população adulta brasileira passou de 18,7% (IC95%: 18,0-19,3) em 2013 para 22,3% (IC95%: 21,7-22,9) em 2019 ^39^. Outros estudos realizados em vários locais do Brasil apresentaram estimativas que variaram de 10,9% a 41,5% ^40^ ^,^ ^41^ ^,^ ^42^ ^,^ ^43^ ^,^ ^44^. Portanto, este estudo mostra que a prevalência encontrada (51,4%) é superior àquela observada na população geral brasileira ^39^ e em populações específicas.

O achado deste estudo está de acordo com estudos realizados em populações nativas de outros países, como Austrália e Canadá. Randall et al. ^28^, ao comparar indígenas com não indígenas na Austrália, encontraram maior probabilidade de multimorbidade entre os indígenas (2,59; IC95%: 2,55-2,62) quando comparados aos não indígenas. Também na Austrália, verificou-se maior prevalência de multimorbidade entre os aborígenes (24,2%) em comparação com os não aborígenes (20,7%) ^45^. No Canadá, Kuwornu et al. ^46^ encontraram maior prevalência de multimorbidade entre o grupo indígena (38,9%; IC95%: 36,5-41,3) quando comparado aos não indígenas (30,7%; IC95%: 28,9-32,6).

No Brasil, não foram encontrados estudos que avaliam a prevalência de multimorbidade especificamente na população indígena até o momento dessa pesquisa. O *Primeiro Inquérito Nacional de Saúde e Nutrição dos Povos Indígenas* ^9^, realizado em 2009, foi um importante marco para a saúde dos povos indígenas brasileiros. Entretanto, a maior parte dos dados foram provenientes de grupos indígenas da Amazônia e incluiu apenas mulheres e crianças. O trabalho possibilitou compreender a real situação de saúde de mulheres e crianças, apontando para a emergência de DCNT entre as mulheres ^9^.

Em nosso estudo, duas morbidades foram muito frequentes: a obesidade e o diabetes, com prevalências de 37,5% e 19,8%, respectivamente, superando as prevalências correspondentes de 25,9% e 7,7% verificadas na população geral brasileira estudada na PNS em 2019 ^47^ ^,^ ^48^. O incremento das prevalências dessas morbidades em povos indígenas no Brasil vem sendo reportado em vários estudos ^11^ ^,^ ^49^ ^,^ ^50^ ^,^ ^51^ ^,^ ^52^ ^,^ ^53^ e o rápido crescimento dessa condição em indígenas prevê o aumento da incidência de diabetes do tipo 2, o que, aparentemente, já se manifesta na população deste estudo. Cabe lembrar, entretanto, que a natureza transversal do dado não permite inferência causal entre essas condições.

É importante ressaltar que, quando há referência aos povos indígenas no Brasil, deve-se considerar o fato de haver populações residindo em reservas isoladas e populações que vivem em regiões urbanas ou próximas a áreas urbanas ^54^. A população indígena de Aracruz vive em terras já demarcadas, mas mantém forte contato com as populações rurais e urbanas no entorno da reserva. Esse contato faz com que esses indígenas tenham acesso facilitado aos meios de locomoção, uma vez que linhas de ônibus cruzam as aldeias, à alimentação, adquirida em supermercados, e às tecnologias de comunicação, entre outros. Nesse cenário, estudos vêm mostrando o quanto a proximidade dos territórios indígenas com o meio urbano contribui para o aumento da incidência das DCNT entre os indígenas ^10^ ^,^ ^11^.

Ao analisar a prevalência das morbidades de acordo com as etnias Tupiniquim e Guarani, foi identificada diferença significativa na prevalência de obesidade e hipertensão. Entretanto, quando analisada a multimorbidade, a diferença entre as etnias se mantém significativa apenas nas análises brutas. Esse achado pode ser atribuído ao fato de que a diferença foi encontrada apenas nas morbidades que apresentaram maior prevalência na amostra (obesidade e hipertensão).

Entre os fatores associados ao desfecho, o sexo feminino apresentou maior probabilidade de apresentar multimorbidade quando comparado aos homens. Resultados semelhantes foram encontrados em outros estudos realizados no Brasil ^55^ ^,^ ^56^ ^,^ ^57^. Esse achado pode ser explicado pelo fato de as mulheres, neste estudo, apresentarem maior prevalência de obesidade comparada aos homens (46,5% *vs.* 25,2%; p < 0,05 - dados não apresentados em tabelas), sendo essa uma morbidade com forte associação com o diabetes do tipo II e a hipertensão arterial. Mulheres, de fato, como utilizam mais os serviços de saúde, têm maior chance de receber diagnóstico médico ^58^. Não parece ser o caso atual, porque tanto a obesidade como a hipertensão, a diabetes e as dislipidemias foram definidos não por relato, mas por exames. As morbidades referidas foram bem menos frequentes. Além disso, estudos têm associado a maior prevalência de obesidade em mulheres indígenas com o fato de elas realizarem majoritariamente atividades voltadas para o artesanato, o que requer menor gasto energético, quando comparado às atividades laborais dos homens indígenas, que em sua maioria trabalham na lavoura ^49^ ^,^ ^59^ ^,^ ^60^. O viés de sobrevivência, uma vez que os homens apresentam menor expectativa de vida, também poderia explicar essa diferença, mas não parece ser o caso já que a distribuição das idades foi similar entre homens e mulheres, com média ligeiramente menor em mulheres ^61^.

Como esperado, observou-se aumento da prevalência de multimorbidade com o aumento da idade. Uma revisão sistemática, realizada por Violan et al. ^62^, mostrou que a idade foi o determinante que apresentou associação positiva e significativa com a multimorbidade em todos os estudos avaliados. Os mesmos autores explicam que essa associação se deve ao fato de que indivíduos mais velhos estão mais expostos aos fatores estressores ao longo da vida, o que compromete o equilíbrio fisiológico, favorecendo o desenvolvimento de doenças crônicas.

Outro fator associado à multimorbidade foi a escolaridade. Embora, na análise ajustada, a variável escolaridade tenha perdido parte de sua capacidade explicativa, essa redução pode ser atribuída à presença da variável idade. Isso ocorre porque o desfecho estudado é predominante em indivíduos mais idosos, os quais geralmente apresentam menor nível de escolaridade ^63^. O nível de escolaridade é um fator importante para esse desfecho, uma vez que permite que os indivíduos busquem conhecimentos a respeito da promoção da saúde, da adoção de estilos de vida mais saudáveis e, consequentemente, da prevenção de doenças crônicas ^64^.

Os resultados encontrados neste estudo devem ser interpretados no contexto de suas limitações. Entre elas, tem-se o fato de a amostra não ser representativa da população indígena aldeada em Aracruz, pois foi realizada em voluntários, dificultando a extrapolação dos dados apresentados para a população indígena em geral. Entretanto, os dados apresentados muito provavelmente refletem a situação real dos Tupiniquins e Guaranis vivendo na reserva em que o estudo foi realizado. Uma das dificuldades encontradas para se atingir uma amostra mais robusta foi a realização da pesquisa no período da pandemia da COVID-19, fazendo com que alguns não atendessem ao chamamento da pesquisa. Um pequeno viés de retorno ocorreu em relação ao sexo, pois, nas aldeias, a população masculina é de 49,3% e na amostra foi de 42,4%. Em relação às faixas etárias, a amostra é um pouco mais velha do que a população, na qual a frequência de indivíduos de 20-39 anos é de 58,9% e na amostra foi de 50%, enquanto os idosos (≥ 60 anos) são 12,4% da população e 14,4% na amostra. Mesmo assim, pode-se inferir que o quadro geral de morbidades na população seja bastante similar ao descrito na amostra. Além disso, a necessidade de se deslocar para Vitória para a realização dos exames pode ter dificultado a participação das pessoas com limitações para se ausentar do trabalho.

Outra limitação está nas diferenças entre os aspectos de mensuração da multimorbidade, o que dificulta a comparação entre estudos. Ainda, tem-se a utilização de apenas oito morbidades crônicas para contabilizar a multimorbidade, enquanto estudos recomendam a utilização de, no mínimo, 12 morbidades ^65^. Entretanto, destaca-se que, neste estudo, priorizou-se utilizar morbidades que pudessem ser medidas no próprio estudo, quando não diagnosticadas previamente, , favorecendo a força das associações encontradas.

Por fim, tem-se as dificuldades inerentes ao estudo transversal, o que não permite inferir associações de causa e efeito, podendo ter prejudicado a avaliação de algumas associações. Por outro lado, o valor deste estudo está no ineditismo em apresentar a prevalência da multimorbidade numa população indígena brasileira que vive em aldeia com forte relação com o entorno urbano, como ocorre com muitas outras populações indígenas em outros locais do país.

Os achados sobre a multimorbidade na população indígena proporcionam um embasamento importante para análises futuras a respeito da adoção de medidas de prevenção das DCNT, oferta e sustentabilidade do serviço de atenção primária à saúde. Os resultados desta pesquisa mostram uma alta prevalência de multimorbidade nessa população, associada ao sexo feminino, idade e nível de escolaridade, mostrando ser necessário priorizar as estratégias de prevenção nesses grupos.
